# A Non-Chemical System for Online Weed Control

**DOI:** 10.3390/s150407691

**Published:** 2015-03-30

**Authors:** Victor Rueda-Ayala, Gerassimos Peteinatos, Roland Gerhards, Dionisio Andújar

**Affiliations:** 1 Department of Weed Science (360b), University of Hohenheim, 70599 Stuttgart, Germany; E-Mails: g.peteinatos@uni-hohenheim.de(G.P.); roland.gerhards@uni-hohenheim.de (R.G.); 2 Proyecto Prometeo–SENESCYT. General Coordination of Laboratories, Agencia Ecuatoriana de Aseguramiento de la Calidad del Agro, AGROCALIDAD, Vía Interoceánica, km $14\frac{1}{2}$ y Eloy Alfaro, Granja MAGAP, EC170184 Tumbaco, Ecuador; 3 Institute of Agricultural Sciences, Consejo Superior Investigaciones Científicas, CSIC, Serrano 115b, 28006 Madrid, Spain; E-Mail: dionisioandujar@hotmail.com

**Keywords:** selectivity, soil covering, crop-weed-soil variability, fuzzy logic, site-specific harrowing

## Abstract

Non-chemical weed control methods need to be directed towards a site-specific weeding approach, in order to be able to compete the conventional herbicide equivalents. A system for online weed control was developed. It automatically adjusts the tine angle of a harrow and creates different levels of intensity: from gentle to aggressive. Two experimental plots in a maize field were harrowed with two consecutive passes. The plots presented from low to high weed infestation levels. Discriminant capabilities of an ultrasonic sensor were used to determine the crop and weed variability of the field. A controlling unit used ultrasonic readings to adjust the tine angle, producing an appropriate harrowing intensity. Thus, areas with high crop and weed densities were more aggressively harrowed, while areas with lower densities were cultivated with a gentler treatment; areas with very low densities or without weeds were not treated. Although the weed development was relatively advanced and the soil surface was hard, the weed control achieved by the system reached an average of 51% (20%–91%), without causing significant crop damage as a result of harrowing. This system is proposed as a relatively low cost, online, and real-time automatic harrow that improves the weed control efficacy, reduces energy consumption, and avoids the usage of herbicide.

## 1. Introduction

Chemical control has replaced mechanical weeding methods [1]. However, new problems have emerged. Abuse of herbicide has caused appearance of resistant or tolerant weeds [2,3]. Also herbicide residues have been found in food, water, and soil [4]. To avoid these drawbacks, weed management should be directed towards a rational use of herbicides, exchanging chemicals with more environmental friendly methods when possible and trying to balance weed control and yield loss. Non-chemical technologies, such as mechanical weed control, provide an option for controlling weeds without harming the environment, not only in organic but also for conventional farming. For instance, weed harrowing with a flexible-tine harrow can effectively control small broad-leaved weeds, and less effectively deep rooted weeds and grasses [5]. Mechanical weed control tools often perform with a lower efficacy and higher costs than chemical control [6]. Lower efficacy is due to a lower selectivity of the treatments, and because it is the common practice to use the same treatment across the whole field, ignoring the spatial variability of crop and weeds [7]. Therefore, non-chemical weeding methods should improve towards a site-specific approach, in order to be able to compete with conventional herbicide application.

Cultivating tillage with a harrow aims to cover with soil, uproot or tear into small pieces many weed plants, while reducing the effects on the crop [8,9]. The crop damage may increase not only due to the broadcast cultivation with the harrow, but also because in practice farmers apply a constant harrowing intensity throughout the whole field. Adjustment of the intensity in cereals is mostly based on crop growth stage, which may also cause variations in selectivity of harrowing [10,11]. Variations in crop development, weed abundance, and a hard or a loose soil surface affect the harrowing performance, resulting in crop damage and variations in weed control [6,12]. Research during the last decade has shown that it is possible to assess variations in soil density, weed density, and crop development and automatically adjust the intensity using different approaches and prototype systems [12–14].

More aggressive intensity levels are obtained by changing the tine angle in relation to the field surface, increasing driving speeds, or including more than one consecutive passes on the same day of cultivation [5]. Even if increasing speed threw more soil onto cultivated plants, more weed control was not observed [15]. Furthermore, Søgaard [12] argued that it is not possible to achieve optimum driving speeds, when speed is controlled manually on the vehicle. Rasmussen *et al.* [5] found that increasing the number of passes on the same day of cultivation resulted in higher selectivity, specially at low driving speeds. However, it is logical to think that more passes would increase crop damage and more importantly, the operation costs. On flexible-tine harrows, it is possible to adjust the tine angle in relation to the soil surface. Changing the harrowing intensity by varying the tine angle seems the most appropriate way to develop an automated harrowing system. Consequently, the aforementioned automated systems have been designed to adjust the tine angle, thereby varying the harrowing intensity while cultivating the crop.

Søgaard [12] achieved “on-line” automatic adjustment of the intensity based on a principle of maintaining a fixed working depth. However, the author concluded that working depth was not the best way to characterize the harrowing intensity, in terms of weed control. Engelke [13] and Rueda-Ayala *et al.* [14] based their systems on other factors besides soil density, *i.e.*, weed density and crop growth stage. Nevertheless, their prototypes worked mostly off-line using application maps, which were created with data from complex and high-cost sensors such as the photooptic sensors [13] and bispectral cameras [14]. Ultrasonic sensors are robust, low cost devices which have been used in weed science research. Andújar *et al.* [16,17] demonstrated weeds could be detected with ultrasonic sensors, because there was a high correlation of height values with weed plant density and biomass. This study aimed to achieve an online and real-time harrowing system, using the weed detection capability of an ultrasonic sensor and used for real-time characterization of treatment intensity. The objectives were to use the weed density discrimination capability of ultrasonic sensors to automatically control the harrowing intensity in a field experiment, and to test the effectiveness of weed control using the online harrowing system.

## 2. Experimental Section

### 2.1. System Description

This system uses a combination of approaches for characterizing the harrowing intensity: the sensor-based mechanical weed control [14,18] and the measuring-controlling-regulating [13]. Both systems presented some limitations such as the use of complex and expensive sensors for plant identification (e.g., photooptic sensors, differential camera), difficult to calibrate sensors for soil density, and the “laboratory processing” required to generate the application maps, which also indicated the off-line operability. The novelty of the presented system lies on the online assessments of weed abundance with a low cost ultrasonic sensor and real-time adjustment of the tine angle, on the go and in one operation. Variations in crop/weed development and density were considered the deciding factors for harrowing intensity adjustment.

**Figure 1:**
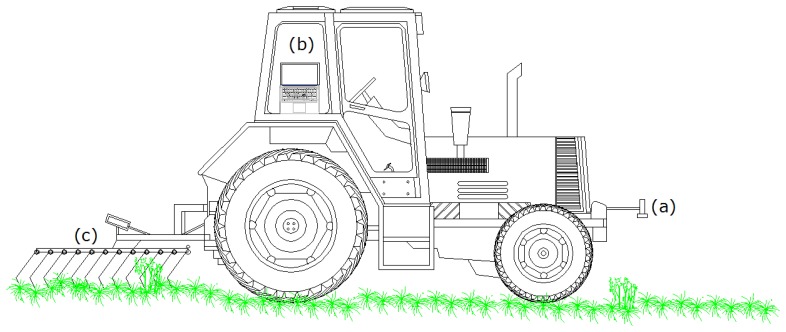
Schematic of the tractor with the three discrete subsystems: (**a**) the ultrasonic sensor mounted in front of the tractor; (**b**) the computational unit responsible for gathering the sensor data, interpreting them, running the decision making algorithm and controlling the actuator; and (**c**) the harrow actuator

The mechanical part of the system for automatic harrowing presented in this paper was based on principles of a previously developed system [14]. The current system was composed of: (i) an ultrasonic sensor mounted in front of a tractor, responsible for measuring the plant height; (ii) a small portable computer which gathered the sensor data, interpreted them, decided about the harrowing intensity and sent the commands to the actuator; and (iii) a flexible tine harrow, where the angle of the tines was changed (Figure 1). Each of the subsystems will be described in the following paragraphs.

#### 2.1.1. Functioning of the Ultrasonic Sensor

Discriminant capabilities of an ultrasonic sensor Pepperl+Fuchs UC2000-30GM-IUR2-V15 (Pepperl+Fuchs GmbH, Mannheim, Germany) were employed to measure the crop and weed variability. Ultrasonic devices measure the distance of emitted-reflected sound waves. The sensor produces a short bust of sound in a unique direction and waits its return after impacting an object. The signal aims the surface of crop-weed-ground mixture and reflects an echo from the various leaves, representing a specific circular field of view. This distance is measured according to the time of flight of the acoustic signal emitted by the sensor, which is therefore transformed into a voltage signal. This voltage can be converted into distance units, based on predefined calibration of the sensor.

In order to increase the measuring accuracy, the specific sensor offers the ability of calibration and arrangement of the minimum and maximum distance measured. Minimum distance measured can be 0.08m and the maximum 2 m, with a minimum accuracy of 0.002 m. The transducer ultrasound frequency is approximately 180 kHz. The time lapse between emission and reception of the signal is the time needed to cover the distance twice. Using the speed of sound, the distance to leaf and ground obstacles was calculated with Equation (1),

(1) $$R=\frac{T_{L}}{2}\times s$$

where *T_L_* is the time of flight since the signal was sent until it was received back and *s* is the speed of sound ( $\sim 342\frac{m}{s}$).

The actual distance measured by the sensor to the plant leaves was expected to highly correlate with both, the plant heights at the measuring point and the plant density [16]. Shorter distances assessed meant higher (weed) plants (cm), and ultrasonic height measured proportionally correlated with plant density, *r* > 90% [17]. In our study, the ultrasonic sensor was mounted on the front of a tractor at 0.7 m above the ground, pointing vertically downwards. Plant height assessments were acquired on the field prior to the harrowing application. Similarly, all plant height assessed with the sensor highly correlated to plant densities.

The plant height was calculated by subtracting the measured distance from the predefined mounting height of the sensor (0.7 m). The sensor was calibrated to measure distances between 0.1 and 0.75 m from its mounting point. This specific values were chosen in order for the sensor to be able to take measurements from 0.05 m below the ground level until a maximum height of 0.6 m. The possibility of negative values was implemented in order to avoid sensor errors, due to distances higher than 0.7 m, e.g., field irregularities with sallow holes or bare soil patches across the field during harrowing, where the tractor is slightly inclined. The above mentioned measuring scheme, the sensor's accuracy was 0.008 m. These settings proved to be sufficient for the current experiment. The output was an analogue DC voltage (0–10 V) with a 10 Hz sampling rate that represented these distances. The voltage output of the ultrasonic was measured and converted to digital values with a Labjack U12 data acquisition (DAQ) card (LabJack Corporation, Lakewood, CO, USA).

Ultrasonic sensors have been used for the characterization of plant mass and geometric parameters in horticulture [19,20]. Moreover, they have proven to function with arable crops. This sensor is well suited for field conditions, thanks to its internal temperature correction to avoid malfunctioning of the electronics, suitable physical protection and high resistance to vibrations.

#### 2.1.2. Controlling System

A small portable computer (Asus Eee PC 901) was used to gather the sensor data, interpreted them, decided about the harrowing intensity and sent the commands to the actuator. Voltage data from the ultrasonic sensor with a frequency of 10 Hz were gathered through the DAQ card. Based on the predefined calibration (0.1 to 0.75 m) the measured plant height was calculated with Equation (2):

(2) $$\text{Height}=0.75-\left(V\times Cal_{\text{factor}}+0.1\right)$$

where *Height* is the calculated height in m, *V* is the voltage measured in V and *Cal_factor_* is the calibration factor. For the current setup *Cal_factor_* equalled to 0.065. The ability to change the calibration setup, in order to fit different tractor and measurement profiles has been taken into account. In order to attenuate high frequency fluctuations inside the ultrasonic sensor's voltage signal, a low pass filter was implemented via software in the raw voltage data. This filter was based on the tenth measurement moving average, giving each time the mean value of the current and the nine previous measurements (the last 1 s). This filter suppressed high alternations produced from mechanical vibrations, electric noise, and field obstacles. However, a small delay occurred in shift plant density changes of about 0.5 s. Based on the application speed of 12 km h^−1^this can be translated into a 2 m classification shift, with repercussions presented and discussed onwards.

Based on the height measurements of the ultrasonic sensor, we designed a Decision Support System (DSS) to adjust the harrowing intensity. The principle of the DSS was, that areas of large plant densities were assumed to originate from higher weed/total-plants ratios, thus requiring more aggressive treatments. Conversely, areas of smaller densities needed gentler treatments, because they derived from lower ratios. Areas of very low weed densities or without plants did not require any cultivation with the harrow.

The harrowing intensity adjustment was based on weed density assessments and tine angles previously tested to successfully control those weed infestation levels [14]. Fuzzy logic was used to define the classes for weed densities which correlated to ultrasonic height measurements. A fuzzy set of the weed density *I_WD_* was created, after correlating data of ultrasonic readings (height) with weed densities measured in the laboratory. Harrowing intensities were correspondingly increased, as the weed density increased as well. Furthermore, crop plant height fairly described the total plant density situation (crop and weeds); it was assumed a uniform crop plant height across the field. At the moment of harrowing, weeds were small and contributed insignificantly to the total plant coverage. Therefore, any increment in a height reading would mean the presence of weeds.

In order to adjust the intensity to the actual field's needs, the application rate was linearly managed. We started the sensor calibration using the predefined weed density classes determined for cereals by Rueda-Ayala [6], Rueda-Ayala *et al.* [14]: “none”, “low”, “medium”, “high”, with 0–15, 16–42, 40–63, and >60 plants m^−2^, respectively. These weed density thresholds were applied, since for maize the thresholds for weed control have only been defined for herbicide application [21,22]. However, after gathering measurements with the ultrasonic sensor in the experimental field, five weed density classes were fine-tuned to correspond with the five harrowing intensity classes (Table 1), which the implement can generate [14]. The incoming voltage signal, derived by the ultrasonic sensor was translated into height distances and the corresponding plant densities. Furthermore, the intensity was micro-adjusted between each level.

**Figure 2:**
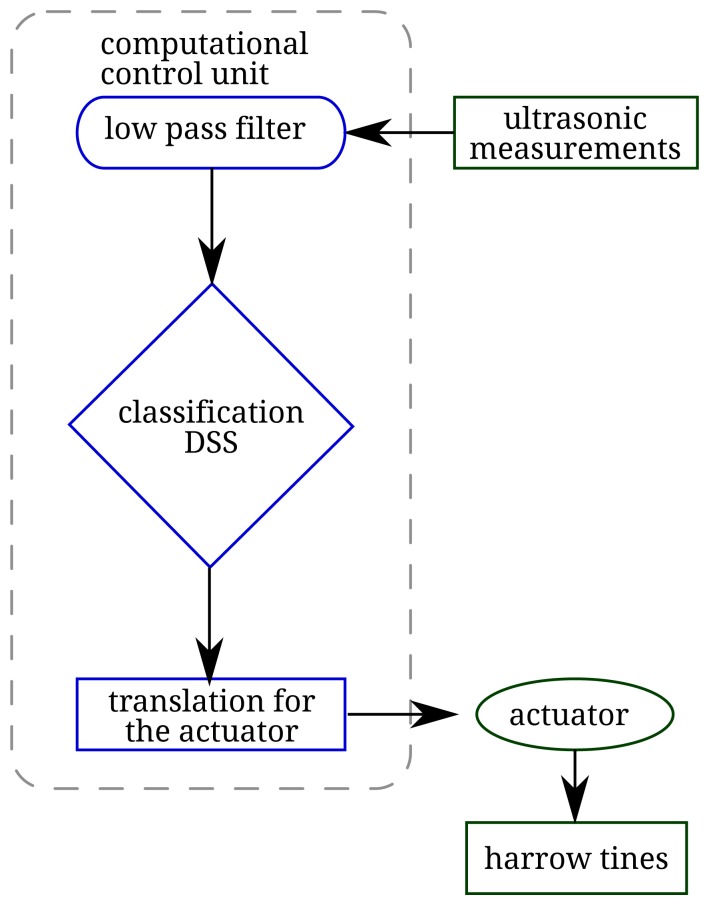
Flow chart of the various elements which composed the complete experimental setup, from measurement to the final adjustment of the application rate.

Inside the control unit, the DSS separated input height values into five discrete classes -five plant height classes (Figure 2; Table 1). Each of these classes represented the reference plant height (crop & weeds), treated with its corresponding harrowing intensity (tine angle). Each ultrasonic measurement was related to the corresponding intensity class, then the control unit activated the electric actuator to move the harrow tines, online. For each input value residing into a specific plant height class, a ratio was calculated to find its specific distance inside by Equation (3).

(3) $$R=\frac{S_{\text{height}}-C_{\text{minh}}}{C_{\text{maxh}}-C_{\text{minh}}}$$

where *R* is the calculated ratio, *S_height_* the height value received by the ultrasonic sensor, *C_minh_* and *C_maxh_* are the minimum and maximum height assigned for a specific class, respectively. This ratio was used to accordingly specify the harrow intensity, after the predefined intensities [6,14], Equation (4).

(4) $$HI=R\cdot \left(C_{\text{maxhi}}-C_{\text{minhi}}\right)+C_{\text{minhi}}$$

where *HI* is the harrow intensity and *C_minhi_* and *C_maxhi_* are the minimum and maximum harrow intensity, respectively assigned for this specific class.

In this DSS the first class represented the height attributed to the average crop density. Therefore, for all input values below this height the harrowing intensity was set to be “none”. It was assumed that weeds were absent, or the weed density was too low to compete with the crop. The rest of the classes represented the plant height span for low, medium and high weed infestation levels. These weed infestation levels were treated using a “lightest”, “light” or “strong” intensity. When the weed infestation was too high, *i.e.*, weed plants were even bigger than the crop, the harrowing intensity was set to be very aggressive: “strongest”.

The DSS was also designed to be flexible for fine-tuning by the use of more classes, each with its corresponding harrow intensity. If the weed infestation is highly variable throughout the whole field, more classes can be defined, thus more precise harrowing intensities can be applied, site-specifically. Although each assessed height had to be uniquely identified inside a class, the harrowing intensity level could overlap between different classes. Therefore, the DSS grouped different weed infestations into similar harrowing treatment. In order to check the performance of the system, all data were stored every two seconds during the harrowing operations.

#### 2.1.3. Flexible-Tine Harrow

The implement used was a 6m wide flexible-tine harrow (Hatzenbichler Austrian Agrotechnik). This harrow consisted of four autonomous subunits, each of 1.5 m width. A subunit was composed of six rows of tines, each containing eight tines, distant 0.2 m from each other. All six rows were mechanically connected to be moved together back and forward by an actuator, which changed the tine angle for cultivating the soil. The actuator was an electric cylinder that could expand 0.115m in a total time of 7.5 s. All actuators were adjusted by a controlling unit that received data of the ultrasonic weed assessments and sent the signal to the actuators to change the tine angle.

**Figure 3:**
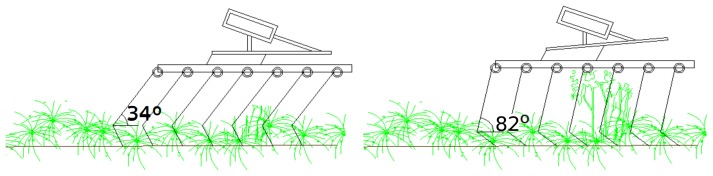
Schematic of the tine angle used, creating a low harrowing intensity (left) and a high harrow intensity (right).

The controlling unit had 8-bit resolution, resulting in 256 different tine angles (harrowing intensities). However, the controlling unit worked flawlessly only until 90% of the actual capacity, which allowed generation of maximum 90% harrowing intensity. Irregularities in the technical part of the system prohibited the use of full harrowing capacity, *i.e.*, 90° tine angle. In order to avoid 100% utilization of the harrow, the last plant height class was artificially increased by 10% (Table 1). Further development in the DSS could set a predefined upper harrow limitation, which was not present at the time of application. In consequence, the actual tine angle was changed from 34° (gentle harrowing intensity) to 82° (aggressive harrowing intensity) as presented in Figure 3 [23]. The presented system included the direct transmission of the ultrasonic signal to the controlling unit, thus resulting in the online and real-time intensity adjustment.

### 2.2. Experimental Site and Measurements

A field experiment in maize (*Zea mays* L.) was implemented during spring-summer 2013. The experimental site was located in a field of the University of Hohenheim, at the experimental research station Ihinger Hof (48°45′ N, 8°56′ E), Renningen. Maize was sown at a row distance of 0.7 m, on 20 May, 2013. For the examination of the system, two experimental plots were harrowed. Each plots had a length of approximately 50 m, and was harrowed with two consecutive passes in the same day. Two passes were used, due to the highly variable weed infestation levels and the very hard soil surface condition. One plot had higher weed infestation and plant height levels than the other; it was intended to identify whether the ultrasonic can cope with extreme variability. Assessments and harrowing operations took place on 7 June, when weather conditions were dry.

Weed abundance classes and harrowing intensities were created based on a ultrasonic readings collected during the previous year, and at the same day, prior to the experiment. Calibration of the control algorithm was done before harrowing, using in-field measurements, randomly distributed across all experimental plots. Five weed infestation classes were obtained (Table 1), and for each class a corresponding harrowing intensity was used. The ultrasonic sensor was mounted on a frame in front of the vehicle, approximately 7.5 m ahead of the implement, to provide sufficient time for adjusting the tine angle. Driving speed for harrowing was 12 km h^−1^. The computational time from receiving the data to adjustment of the actual decision was less than 100 ms, giving more than 2.2 s for the electric actuator to move the tines to the forthcoming intensity level. This configuration permitted a ±30% shift for subsequent tine angle changes. Within this threshold, the implement could adjust the tine angle on time.

Accuracy of the measuring-actuation system was tested by means of digital images and visual assessments. Digital images were acquired with a camera at 12 sampling points in each plot, prior and after the application. An 8 megapixel CCD camera equipped with a Nikkor lens was used to capture the images. During image taking, the camera was hand-held at about 1.30m height and pointing vertically downwards. Images were taken on the inter-row area covering a circle of 0.2m diameter. From these circles, leaf cover index was calculated through digital image analysis [18]. For each pixel of the images the Effective Greenness index was calculated. Based on a predefined threshold the pixels were classified as plant or no-plant (soil). Leaf cover index was calculated as the ratio of plant to soil pixels per image. In each measuring point, crop and weed plants were counted prior and after harrowing.

Dominant weed species, found in the field, were visually determined: *Chenopodium album* L., *Polygonum convolvulus* L., *Galium aparine* L., *Echinochloa crus-galli* (L.) P. Beauv.and *Veronica persica* Poir. Weed growth stages ranged from BBCH 9 to BBCH 15–16 on the first sampling date and BBCH 9 to BBCH 23–24 on the second date [24]. The relationship between weed density and biomass with ultrasonic readings was assessed at those two dates. A number of 130 samples was taken. The field covered a broad variation of weed composition: grasses, broad-leaved weeds and mixtures of both. Plant height measurement with the ultrasonic sensor served to relate each sampling point to the corresponding weed densities, on-the-go, and harrowing intensity was adjusted in real-time through an inference system.

## 3. Results and Discussion

**Figure 4:**
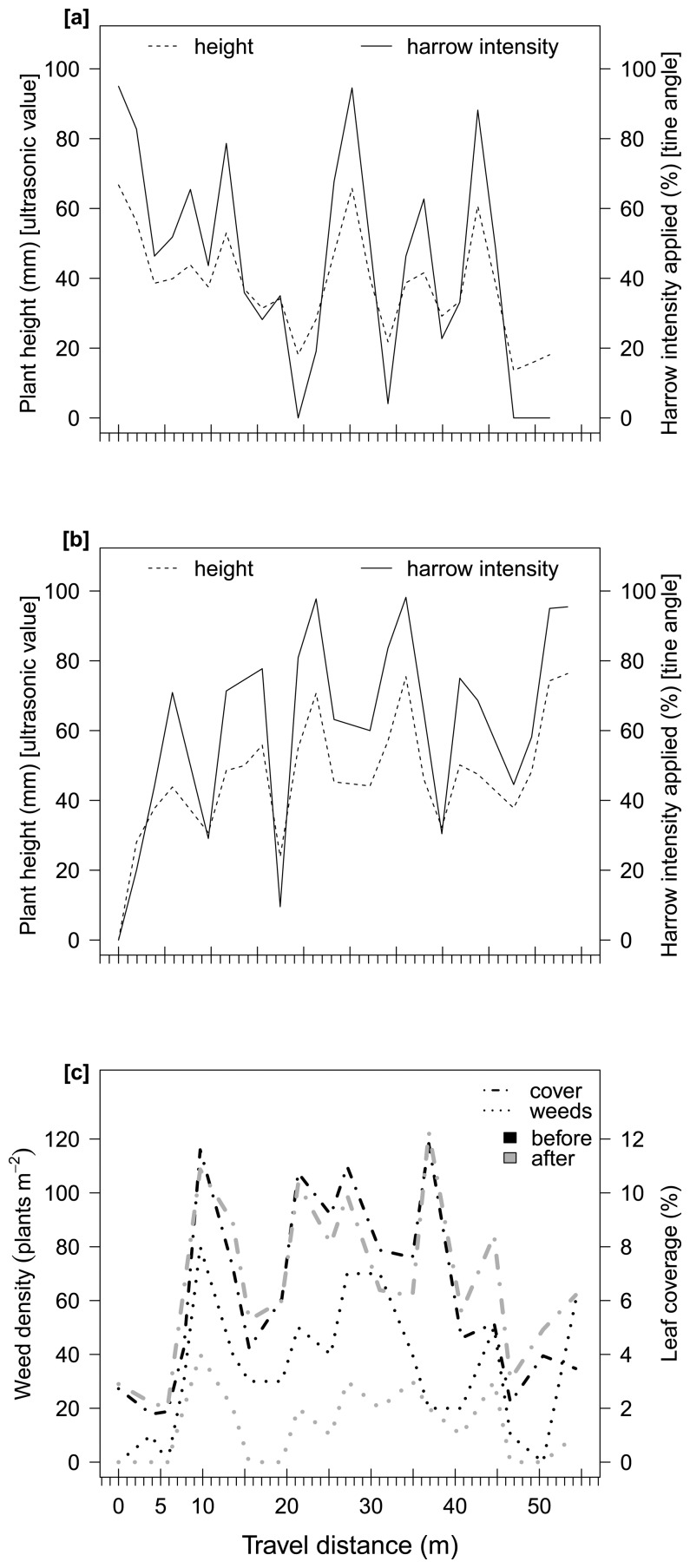
Ultrasonic value of plant height and harrowing intensity applied by the control unit in experimental plot A, first pass (**a**) and second pass (**b**); and variation in weed density and leaf cover before and after harrowing (**c**).

**Figure 5:**
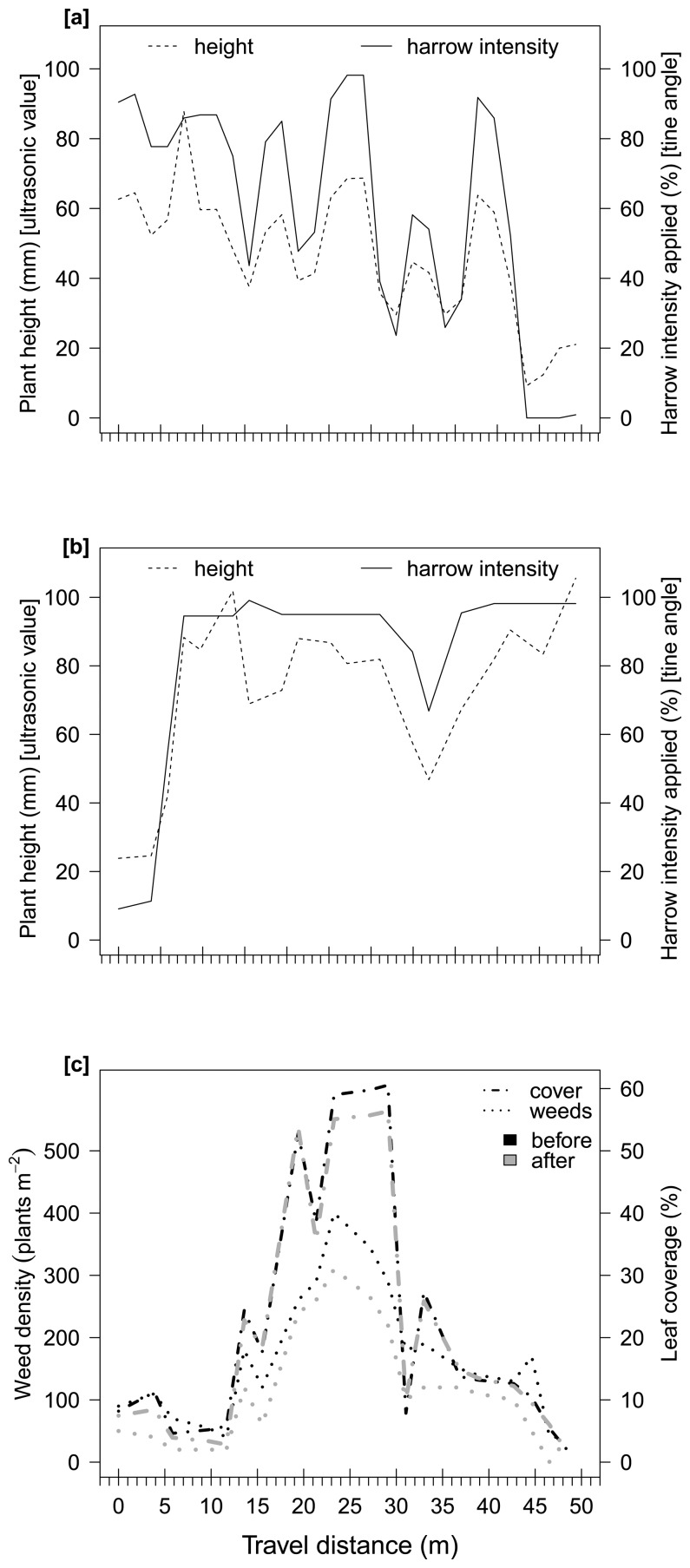
Ultrasonic value of plant height and harrowing intensity applied by the control unit in experimental plot B, first pass (**a**) and second pass (**b**); and variation in weed density and leaf cover before and after harrowing (**c**).

Weed densities in the experimental plots varied, from 0 to 400 plants m^−2^. The most dominant species (percent of the total density) were *Chenopodium album* L. (40%), *Polygonum convolvulus* L. (30%), *Galium aparine* L. (10%), *Echinochloa crus-galli* (L.) P. Beauv. (10%), *Veronica persica* Poir (5%) and other species (5%). Weed plants were in between BBCH growth stages 12 to 30 and maize plants were nearly at 4-leaf stage. Based on the weed counts before and after harrowing, the weed control was calculated from 22% to 90% (average 51%) in both plots (Figures 4 and 5c). The general leaf coverage, including crop and weed plants, was reduced due to harrowing by 4% in average and 15% maximum. Thus, the crop was very little disturbed or insignificantly damaged.

In Figures 4 and 5, the plant heights, measured by the ultrasonic sensor, represent the weed infestation, existent before harrowing. The harrowing intensity sent by the control unit to the tines in order to change their angle –thus adjusting the harrowing intensity– corresponded well to the change in weed infestation level along the field. Figure 4a shows that the harrowing intensity in the first pass corresponded fairly well to the assessed plant height by the ultrasonic sensor. The Pearson's correlation was determined with an *r* = 0.99. Although in Figures 4b and 5a, b there was a small delay of maximum 2 m between the ultrasonic value and the harrowing intensity applied. This delay can be expected. Input of the ultrasonic sensor passed through a low pass filtering system, which normalized the high frequency values measured. This filter produced a slowing down effect of actual rapid changes of ∼0.5s. Taking that into account, along with the driving speed used in the current experiment (12 km h^−1^), this 2m variation is the spatial representation of the above delay. The Pearson's correlation, for the three aforementioned cases, were calculated as *r* = 0.96, *r* = 0.95 and *r* = 0.88, respectively.

**Figure 6:**
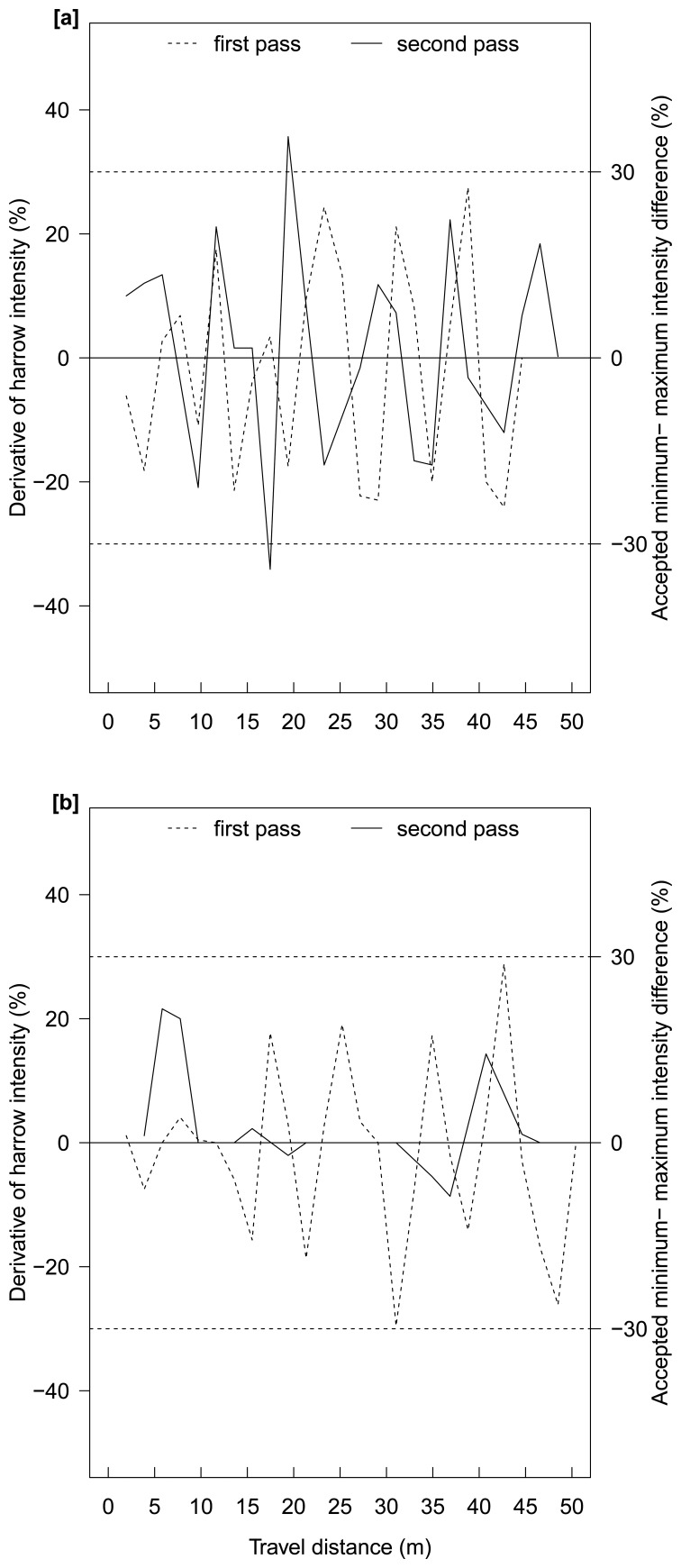
Harrow intensity adjustment needed per time (s) on the first and second pass of Plot A (**a**) and Plot B (**b**). A 30% threshold was accepted to properly adjust the tine angle at 12 km h^−1^ driving speed.

In Figure 6, the first derivative of harrow intensity needed is presented. Based on the current setup, the tine angle was timely adjusted when the harrow intensity derivation was different ±30% of the total intensity. This precondition prevailed across almost the whole experiment, except where a fluctuation of −32% and +32% occurred, exactly one after the other. This fluctuation was encountered only once, as the needed adjustment slightly exceeded the 30% threshold. Consequently, the system was found to perform satisfactorily, even at high driving speeds (e.g., 12 km h^−1^).

Søgaard [12] mentioned that the most important limitation for the actuator occurred when the tine angle was at its maximum or minimum. Thus, at both angles the actuator remained static for a short period, even if the measured variability required a change. Furthermore, this delay was repeated at driving speeds of 2, 6 and 10 km h^−1^. The system presented here has two advantages: (i) a delay did not occur in the actuator when the applied intensity was zero (none) or maximum (strongest); and (ii) the distance between the ultrasonic sensor and the harrow was sufficient to get a full tine angle adjustment on time at high driving speeds. Therefore, the applied harrowing intensity fitted the weed infestation variability well (Pearson's correlation coefficient > 0.85), and was accurately changed, site-specifically (Figures 4a,b and 5a).

Figures 4c and 5c show the weed infestation and leaf coverage in the fields before and after harrowing. In Plot A, the weed density was predominantly in between 100 and 400 plants m^−2^ (Figure 4c), while in plot B it occurred between 20 and 80 plants m^−2^. Weed plants were bigger in plot A than in plot B.

Apparently, the ultrasonic sensor shows a drawback when measuring plant height, since height is inversely correlated with the measured distance. Depending on the soil resistance to the cultivation with the harrow, a backward force can be generated, tilting the vehicle while driving. The first pass with the harrow was on hard soil conditions, which increased the backward force that slightly tilted the tractor. As a result, the measured distance between the ultrasonic sensor and the plants appeared bigger, and an underestimation of the plant height (thus plant density) occurred. In both plots, greater plant height values were assessed by the sensor in the second pass (*i.e.*, shorter measured distance). The soil was a little loosened after the first cultivation, which may have reduced the soil resistance to harrowing, thereby reducing the tilting effect. As a consequence, plant height in the second pass appeared to represent higher plants than at the first pass, and therefore strong harrow intensities had to be applied for the second pass.

Fusion of the ultrasonic sensor with a gyroscope could compensate the inefficiency of the aforementioned problem. Moreover, in this study another possible solution was tested. Based on the height and its correlation with weed density, different harrowing intensities were applied in the two different plots. In plot B, where the plant density exceeded 60 plants m^−2^, harrow intensity was predominantly the aggressive treatment possible. The median was 75%, with a mean of 60% for the first pass, and 95% with a mean of 83% for the second pass. Since most of the measurements lay in the last two DSS's classes (3 and 4), the fluctuation was small. Consequently, the system performed satisfactorily although the implements' limitations.

In plot A, the weed density was lower and representations of all 5 classes could be observed. The harrow was also adjusted, based on this weed density, reducing the harrowing intensity. The mean and median were 45% in the first pass and 63% in the second pass, respectively. Because in plot A all weed classes defined in the DSS were represented, the intensity fluctuation was higher. Even though the first pass provided higher frequency alternations, the intensity fluctuation never exceeded ±28%. In the second pass there was a threshold infringement of ±32% intensity increase, which surpassed the systems limitations. This abrupt change in harrow intensity might be the result of a crop free area (gap). To counteract the threshold infringement, an implement with faster angle change capability may be required, otherwise the driving speed should be reduced. In this study, if the driving speed was reduced to 10 km h^−1^, the system threshold would have increased to 35%. Thus all intensity fluctuations would fit that threshold.

The system presented here has shown some advantages, compared with the one described in Rueda-Ayala *et al.* [14]. In Rueda-Ayala *et al.* [14] the harrowing intensity adjustment algorithm worked off-line, based on application maps created in the laboratory after image processing to determine weed abundance from differential images. The differential camera that takes two images, one in the infrared range and the other in the red range, is a relatively expensive sensor (nearly 47,356.03 USD on 11 March 2015 [25]). Through image analyses, weed maps and harrowing application maps were constructed in the laboratory. The control unit received that information from application maps to action the actuator and adjust the tine angle site-specifically, and with the aid of a DGPS for correct possitioning. Furthermore, the driving speed of the harrow for optimal weed control was lower than 10 km h^−1^.

For future experiments to validate the system, it must be tested at earlier crop growth stages (e.g., BBCH 11 to 16). More importantly, the weed population should also be at young growth stages (*i.e.*, close to the cotyledon stage), in order to fully profit of this online automatic harrowing system.

## 4. Conclusions

The present study proposes an online and real-time mechanical weed control system. Variations in weed and crop density were assessed with an ultrasonic sensor, which is a low cost sensor (nearly 638.50 USD on 11 March 2015 [26]). All measurements were taken on-the-go, across two experimental fields. Voltage signals were acquired and sent online, directly to a control unit. This control unit ordered actuators to move and adjust the tine angle, site-specifically according to the measured plant density variations, in real-time and in one operation. Positioning for the measurement-treatment operation was based on distance from the sensor to the actuator, and the reaction speed of the actuators, thus not requiring a GPS. The system performed well at high driving speeds needed for harrowing operations (e.g., 12 km h^−1^).

This system offers a relatively low cost, automatic, online harrowing alternative. Under the correct conditions, it will improve the efficacy of weed control and reduce the energy consumption of the vehicle. Furthermore, this system could be used to detect weeds smaller than the crop and areas with patches of perennial weed species (e.g., *Cirsum arvense* L.) or areas without a crop, because these are applications required to direct machines to areas where weed control methods are warranted.

## Figures and Tables

**Table 1:** Ranges of measured plant height in the field experiment, which correspond to each of the five discrete classes in the Decision Support System to control the harrowing intensity.

**Class**	**Min. Height (cm)**	**Max. Height (cm)**	**Plant Density (plants m ^−1^)**	**Harrowing Intensity**
0	0	10	0–15	none
1	10	15	16–30	lightest
2	15	20	28–47	light
3	20	25	45–63	strong
4	25	77 ^†^	>60	strongest

† The actual maximum plant height to be measured was 70 cm, but 77 cm were artificially implemented due to technical irregularities, see Section 2.1.3.
